# A simple and effective method to encapsulate tobacco mesophyll protoplasts to maintain cell viability^☆^

**DOI:** 10.1016/j.mex.2014.11.004

**Published:** 2014-11-28

**Authors:** Rong Lei, Wenjie Qiao, Fan Hu, Hongshan Jiang, Shuifang Zhu

**Affiliations:** a Institute of Plant Quarantine, Chinese Academy of Inspection and Quarantine, Beijing, China; b Department of Entomology, College of Agronomy and Biotechnology, China Agricultural University, Beijing, China

**Keywords:** Protoplast immobilization, Tris, tris(hydroxymethyl)aminomethane, MES, 2-(*N-*morpholino)ethanesulfonic acid, PET, polyethylene terephthalate, ETAF, extra thin alginate film, TAL, thin alginate layer, FDA, fluorescein diacetate, CLSM, confocal laser scanning microscope, Protoplasts, Immobilization, Silica sol–gel, Alginate, Thin layer

## Abstract

Protoplasts have been widely used for genetic transformation, cell fusion, and somatic mutation due to the absence of a cell wall. However, without the protection of a cell wall, protoplasts are easy to rupture and aggregate during washing, collecting, and gene transfection. In this work, we propose a simple and effective silica/alginate two-step method to immobilize protoplasts with advantages in experimental manipulation and microscopic imaging, as well as in potentially studying cell biological processes such as secretion and metabolism. The proposed two-step immobilization method adopts Transwell with clear tissue culture-treated membrane to support protoplasts in the form of uniform thin layer, which has three unique properties.

• The tissue culture-treated membrane has a good affinity for the plant cell; thus, protoplasts can spread evenly and form a very thin layer.
• There are more choices for membrane pore size, depending on the application.
• It is very convenient to change or collect the solution without mechanically disturbing the protoplasts. This simple and effective silica sol–gel/alginate two-step immobilization of protoplasts in Transwell has great potential for applications in genetic transformation, metabolite production, and migration assays.

## Graphical abstract

**Figure:**
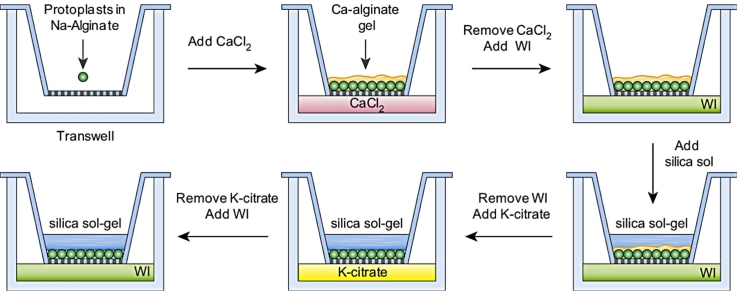

## Method details

In this silica/alginate two-step immobilization procedure, we use Transwell as a support for immobilized protoplasts, which has three unique properties. (1) The tissue culture-treated membrane has a good affinity for the plant cell; thus, protoplasts can spread evenly and form a very thin layer. (2) There are more choices for membrane pore size, depending on corresponding applications. (3) It is very convenient to change or collect the solution without mechanically disturbing the protoplasts. We hope this simple and effective silica sol–gel/alginate two-step immobilization of protoplasts in Transwell will have great potential for applications in genetic transformation, metabolites production and migration assays.

### Preparation of the plant protoplasts

#### Materials

• Tobacco plant *Nicotiana tabacum* cv. White Burley, grown in a temperature and moisture-controlled growth chamber with a 12 h-light/12 h-dark cycle at 24–27 °C, RH 75%.
• Celluase “ONOZUKA” R-10 and macerozyme^®^R-10 were purchased from Yakult Pharmaceutical Industry Co., Ltd. (Tokyo, Japan). 2-(*N*-morpholino)ethanesulfonic acid (MES), mannitol, potassium citrate, fluorescein diacetate (FDA), sodium alginate, sodium silicate, acid ion exchange resin (Amberlite^®^ IR120), LUDOX^®^ HS-40 colloidal silica (40 wt.% suspension in water), tris(hydroxymethyl) aminomethane (tris), PEG 4000 (Fluca, cat. no. 81240) and succinic acid were obtained from Sigma–Aldrich. KCl, NaCl, and CaCl_2_were purchased from Beijing Chemical Works (Beijing, China). 12 mm polyethylene terephthalate (PET) membrane with 0.3 μm pore size transwell-clear inserts (Costar^®^) was purchased from Corning (Corning, USA). All solutions were prepared using Milli-Q water (resistivity = 18.2 Ωcm).

#### Reagent setup

• The pH of 0.2 M MES solution was adjusted with 1.0 M tris solution to 5.8.
• Protoplast medium (PM): prepare 20 mM MES (pH 5.8) containing 0.5 M mannitol, 20 mM KCl, and 10 mM CaCl_2_.
• Enzyme solution: prepare 1% (wt/vol) cellulase Onozuka R-10 and 0.5% (wt/vol) macerozyme R-10 in PM solution. Warm the solution at 55 °C for 10 min to enhance solubility. Cool to room temperature (25 °C). Filter the final enzyme solution through a 0.22-μm syringe filter device into a Petri dish (100 × 25 mm^2^ for 10 ml enzyme solution).
• Washing and incubation solution (WI) solution: prepare 4 mM MES (pH 5.8) containing 0.5 M mannitol and 20 mM KCl.
• FDA stock solution: dissolve 5 mg of FDA in 1 ml acetone, and store in refrigerator.
  *Note*: The protoplasts medium (PM) and the enzyme solution were filter sterilized. The enzyme solution should be freshly prepared.

#### Procedure

**Fig. 1:**
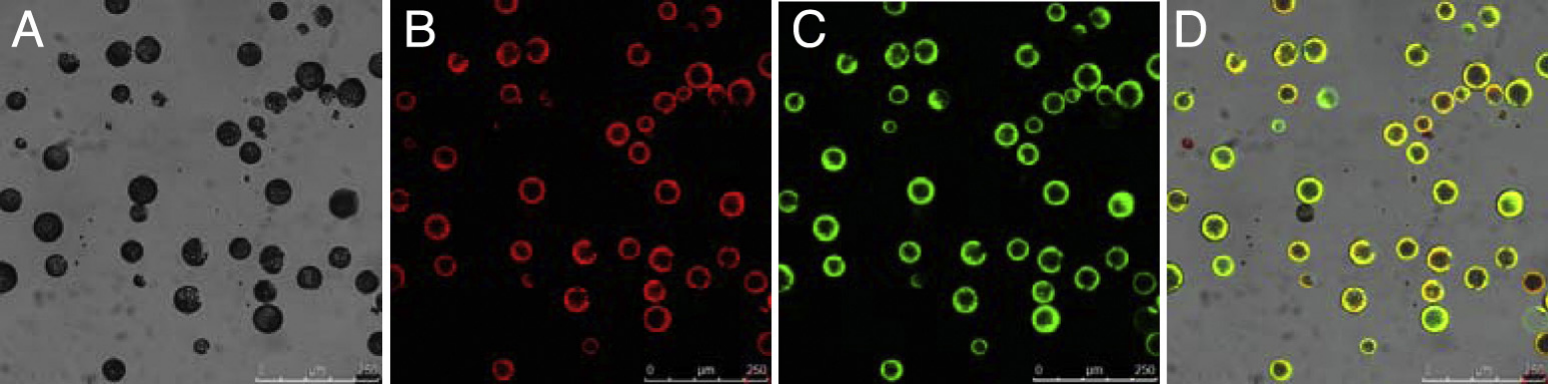
The isolated tobacco protoplasts. (A) Bright field images of tobacco protoplasts; (B) autofluorescence of chloroplasts; (C) green fluorescence of viable protoplasts after FDA staining; (D) merged images of A–C.

The tobacco mesophyll protoplasts used in this study are isolated according to Wu’s method [1]. The plant protoplasts are prepared as follows:

1. Take fully expanded leaves of tobacco plants grown for 40–60 days (pick thick leaves in a similar growth state) to prepare the protoplasts.
2. Excise fully expanded leaves and sterilize by rinsing with 75% alcohol for 10 s and 0.1% mercuric chloride for 4 min, respectively, then wash three to five times with distilled water. Dry off leaves with sterilized filter paper.
3. Remove large vein tissues, pile two to four leaves, and cut only the middle part of the leaves into 0.5–1.0 mm strips using a sharp razor blade without bruising the leaves.
4. Transfer leaf strips quickly and gently into the prepared enzyme (four to six tobacco leaves in 5–10 ml) by dipping both sides of the strips using a pair of flat-tip forceps, incubate the leaf strips in the dark at 25 ± 1 °C for 3.5 h. The enzyme solution should turn green after a gentle spiralling motion.
5. Filter the enzyme solution containing protoplasts after wetting the 100 mesh stainless cell strainer (pore size: 149 μm) with W5 solution, and centrifuge the filtrate in a 50 ml round-bottomed tube at 79 × *g* for 4 min. Remove as much supernatant as possible without disturbing the protoplasts pellet.
6. Re-suspend the protoplasts in 5 ml WI by gentle swirling and centrifuge centrifuged at 79 × *g* for 4 min. Remove as much supernatant as possible and re-suspend the protoplasts in 3 ml WI by gentle swirling, and inject 5 ml of 23% (w/v) sucrose solution into the bottom of centrifuge tube, then centrifuge at 79 × *g* for 4 min.
7. Collect the protoplasts, which is localized in the inter-phase between two solutions into a new 50 ml round-bottomed tube with addition of WI, and centrifuge at 79 × *g* for 4 min. Remove as much supernatant as possible.
8. Re-suspend the protoplast pellet in WI by gentle swirling. Count the protoplast yield by using a haemocytometer under 10× objective microscope. Take 15 μl protoplasts suspension to fill the haemocytometer chamber, and view the protoplasts under a microscope. Focus the microscope on one of the four outer squares in the grid, and count all of the protoplasts in the four 1 mm corner squares. The density of protoplasts was calculated by using the formula of “the average protoplast count per square ×10^4”^. The density of protoplasts was adjusted to 2 × 10^5^ per milliliter of WI.
9. Estimate the density and viability of protoplasts using a haemocytometer by staining the protoplasts with fluorescein diacetate (FDA). Add some amount of the stock solution of FDA to the protoplast medium to obtain the final concentration of 0.01% (w/v).
10. Incubate protoplasts with FDA for 30 min, then view the protoplasts under confocal laser scanning microscope (CLSM) with 10× objective. Set the excitation wavelength of the argon ion laser to 488 nm, and the dual regions of detection between 505–530 nm and 650–730 nm. Viable protoplasts exhibited green fluorescence, accompanied with red autofluorescence of chloroplasts Fig. 1. Count the total number of protoplasts within the red fluorescence field. Observe 10 different views for calculating the average viability.
11. Calculate the viability by dividing the total number of protoplasts by that of viable protoplasts. Perform three times independent calculation, and record the average value as the protoplast yield.
  The microscopic pictures of protoplasts are shown in Fig. 1.

### Immobilization of plant protoplasts using silica sol–gel/alginate

#### Reagent setup

• Sodium alginate solution: prepare 1% (wt/vol) sodium alginate containing 0.45 M mannitol and 4 mM MES (pH 5.8).
• CaCl_2_ solution: prepare 25 mM CaCl_2_ containing 0.45 M mannitol and 4 mM MES (pH 5.8).
• Succinic acid solution: prepare 10% (w/v) succinic acid containing 0.45 M mannitol.
• Potassium citrate solution: prepare 1.0% potassium citrate containing 0.45 M mannitol (pH 5.8).
• “Low-sodium” silicic acid sol: dilute sodium silicate with sterilized ddH_2_O to 1.5 M (concentration of SiO_2_), and eliminate most of sodium ions by using an acid ion exchange resin.
• W5 solution: prepare 2 mM MES (pH 5.8) containing 154 mM NaCl, 125 mM CaCl_2_ and 5 mM KCl.

#### Procedure

1. Mix 50 μl suspended protoplasts (in WI) with 50 μl 1% (w/v) sodium alginate in a Petri dish by gentle swirling.
2. Take 30 μl the above mixture and pave on PET microporous membrane.
3. Add 200 μl of 0.025 M CaCl_2_ solution to the lower compartment under the PET membrane. After 5 min incubation at room temperature, the calcium-alginate thin film was formed. Remove the CaCl_2_ solution, add 500 μl WI solution to the lower compartment to wash the residual CaCl_2_ solution and the calcium-alginate film. Repeat washing three times and remove excess solution with absorbent paper.
4. Mix 200 μl “low-sodium” silicic acid sol with 100 μl LUDOX^®^ HS-40 colloidal silica, and adjust the pH of silica mixture to 6.0–7.0 by using 30 μl of succinic acid solution. Measure the pH by using a pH test paper (Art. No. 92110, MACHEREY-NAGEL, Germany).
5. Pave 70 μl the above aqueous silica sol on the calcium-alginate film and wait for approximately 5 min to let the silica sol became silica soft gel.
6. Add 200 μl potassium citrate solution to the lower compartment, incubate at 4 °C for 30 min.
7. Remove potassium citrate solution and wash Transwell insert with 500 μl WI for three to four times.
8. Add 500 μl WI at the lower compartment and store the immobilized protoplasts at 4 °C.

### Immobilization of protoplasts in extra thin alginate film (ETAF)

#### Procedure

The ETAF technique was based on the method proposed by Pati et al. [2].

1. Mix 50 μl suspended protoplasts (in WI) with 50 μl 1% (w/v) sodium alginate in Petri dish by gentle swirling.
2. Drop 50 μl of 25 mM CaCl_2_ solution containing 0.5 M mannitol and 4 mM MES (pH 5.8) on an autoclaved slide, then place 100 μl protoplasts/sodium alginate mixture over the drop of CaCl_2_. Place a clean autoclaved coverglass immediately and carefully on the top of the drop.
3. Add 50 μl of CaCl_2_ solution from the sides of the coverglass to promote the crosslink of the alginate. Wait for 5 min.
4. After 5 min, gradually remove the coverglass together with the formed extra thin alginate layer containing embedded protoplasts with the help of forceps, and transfer the coverglass into a sterilized Petri dish containing WI.

### Immobilization of protoplasts in thin alginate layer (TAL)

The protocol for plant protoplasts immobilization in the thin alginate layer was presented by Kiełkowska and Adamus [3] with modification. Briefly, suspend 50 μl protoplasts in 50 μl sodium alginate solution and then gently spread onto 1% (w/v) agar containing 25 mM CaCl_2_ to form alginate layer by circular movement of the Petri dish. After 10 min transfer the formed thin layer to a new 6 cm Petri dish containing WI solution.

### Immobilization of protoplasts in Ca-alginate droplets

Mix 100 μl prepared protoplasts suspension with 100 μl sodium alginate in a Petri dish by gentle swirling to obtain a final density of 1 × 10^5^ ml^−1^. Drop the mixture into a 25 mM CaCl_2_ solution containing 0.5 M mannitol and 4 mM MES (pH 5.8) with the aid of a 1.0 ml syringe with a needle (*φ* 0.5 mm). Collect the polymerized droplets using a clean stainless cell strainer (100 mesh, pore size: 149 μm) and transfer the droplets into a 6 cm Petri dish with WI solution.

### Visualization under confocal laser scanning microscope (CLSM)

**Fig. 2:**
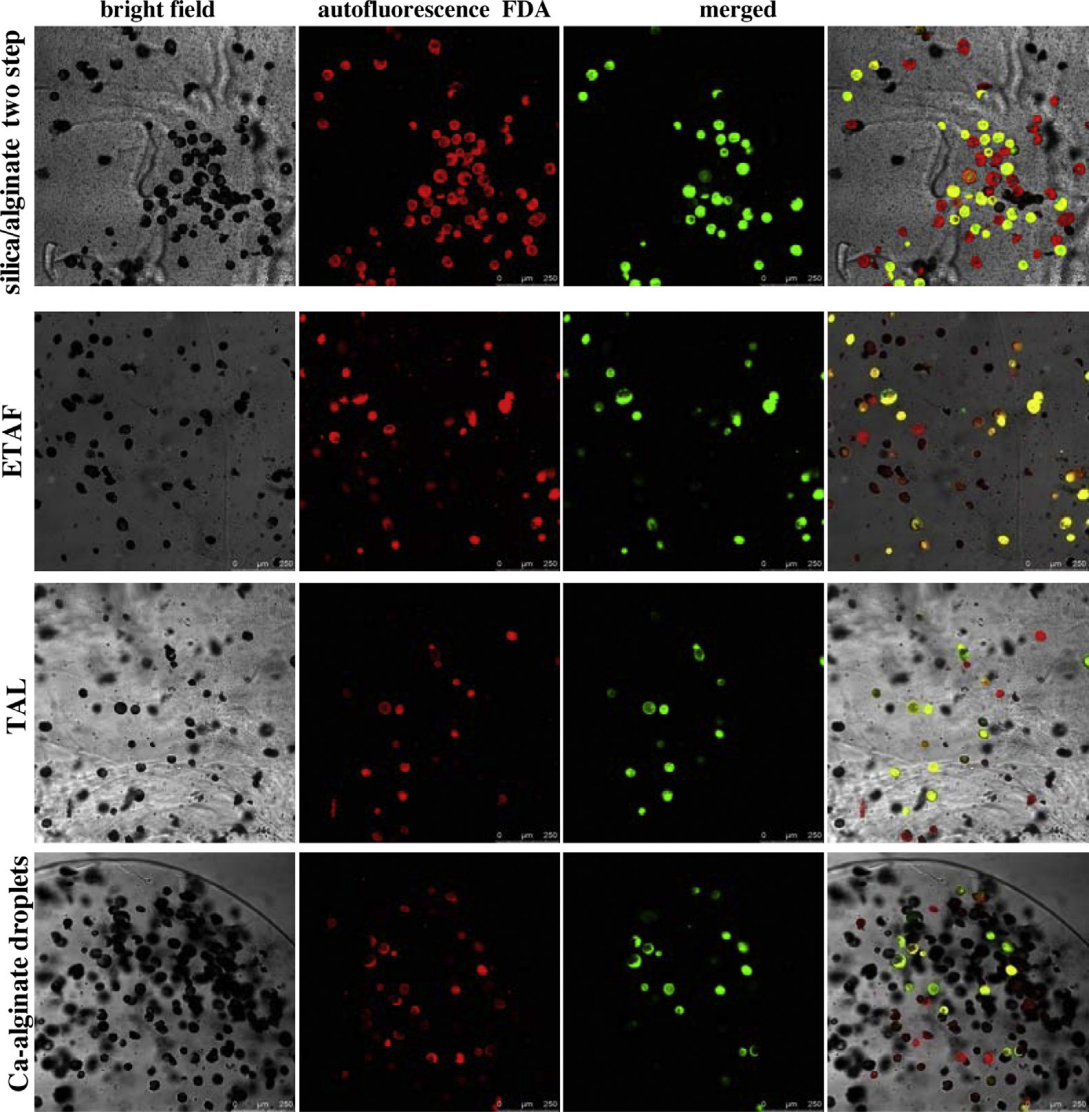
Microscopic pictures of bright field, autofluorescence, fluorescein green fluorescence and merged after FDA staining of protoplasts immobilized by different immobilization methods: (A) silica sol–gel/alginate two-step immobilization method; (B) ETAF; (C) TAL; (D) Ca-alginate droplet immobilization method.

The confocal microscopic pictures of immobilized protoplasts are shown in Fig. 2. By using silica sol–gel/alginate two-step immobilization, most of the immobilized protoplasts distribute evenly on the Transwell membrane and good quality confocal microscopic images are easily obtained, which is advantageous over other preparation methods (Fig. 2). Using the other three immobilization methods, protoplasts are observed and are distributed in the supporting matrix in a multilayer manner such that the majority of the protoplasts are not on the focal plane, making it difficult for cell observation (Fig. 2). As we know, the transparent polyethylene terephthalate (PET) membrane is tissue treated to be hydrophilic, which makes the cell better attached and spread on the surface. Therefore, a small amount of protoplast solution as few as 25–30 μl can be well spread on the Transwell membrane to form a mono-film. With the penetration of CaCl_2_ from the lower pores, Ca-alginate gel is evenly produced on the membrane, without exerting any force on the protoplasts. However, using ETAF to immobilize protoplasts in Ca-alginate gel, we find that pushing the coverglass would easily damage protoplasts and require very clean glasses and good operator skills to yield a success rate of approximately 4 in 10 times. For TAL method, although there is no applied force, the layer is thick and unevenly due to the quick gel formation when the alginate solution contacts with the agar; therefore, the protoplasts cannot be clearly observed in one layer (Fig. 2).

**Fig. 3:**
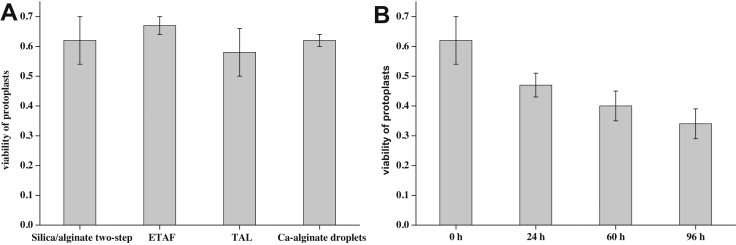
Viability of protoplasts immobilized by four methods (A) and those immobilized in silica sol–gel vs. stored time in 4 °C (B).

The viability of silica sol–gel/alginate two-step immobilization method, ETAF, TAL, and Ca-alginate droplet immobilization method is 0.62 (±0.08), 0.67 (±0.03), 0.58 (±0.08), and 0.62 (±0.02), respectively (Fig. 3A). The viabilities of these four methods are equal in statistical significance. For the silica sol–gel/alginate two-step immobilization method, in addition to the good visualization and uniform distribution, the “low-sodium” silica sol–gel dropped on the Ca-alginate gel can provide a coat for the immobilized protoplasts to further protect them from damage and contamination. With the encapsulation of silica sol–gel coat, the Ca-alginate polymer can be liquefied by potassium citrate if needed. Moreover, it is very simple to transfer the protoplasts and replace the culture media, without repeated centrifugation, thus avoiding risk of crushing the protoplasts. Therefore, the immobilized protoplasts may have versatile applications in the genetic transformation, metabolites production and migration assay.

The protoplasts immobilized in silica sol–gel/alginate without regeneration medium [2], [4] are stored in WI solution at 4 °C in the dark. Because the WI solution cannot provide necessary nutrition for the protoplasts, the viability declines with the time (Fig. 3B), but the immobilized protoplasts maintain a good distribution and shape in a fasting condition.

### Application of immobilized tobacco protoplasts in transient gene expression

#### Reagent setup

• PEG-calcium transfection solution: prepare 40% (wt/vol) PEG 4000 in ddH_2_O containing 0.2 M mannitol and 100 mM CaCl_2_. Freshly prepare PEG solution 1 h before transfection.
• MMG solution: prepare 4 mM MES (pH 5.8) containing 0.4 M mannitol and 15 mM MgCl_2_.

#### Procedure

1. Put the Transwell insert with freshly immobilized protoplasts into 500 μl MMG solution for 20 min to replace the WI solution with MMG solution. Then, transfer the Transwell with immobilized protoplasts to a clean and dry Transwell compartment.
2. Mix 10 μl DNA solution (containing 20 μg of plasmid DNA expressing GFP) and 190 μl MMG solution, and add this mixture to the lower compartment. Shake the Transwell at 80 rpm in incubation shaker for 5 min at room temperature, to make protoplasts enough contact with DNA.
3. Add 110 μl PEG solution into DNA solution, mix completely, and incubate the immobilized protoplasts in the above transfection solution at room temperature in incubate shaker with slow rate of 80 rpm for 15 min.
4. Dilute the transfection solution with 400 μl W5 solution at room temperature to stop the transfection process.
5. Remove the transfection solution in the lower compartment, add 500 μl WI solution to wash the compartment, and remove the WI solution.
6. Add 500 μl WI solution into the lower compartment, and incubate at 25 °C for 20–24 h.
7. View cellular image of GFP under the confocal laser scanning microscope (×100–400).

#### Results

**Fig. 4:**
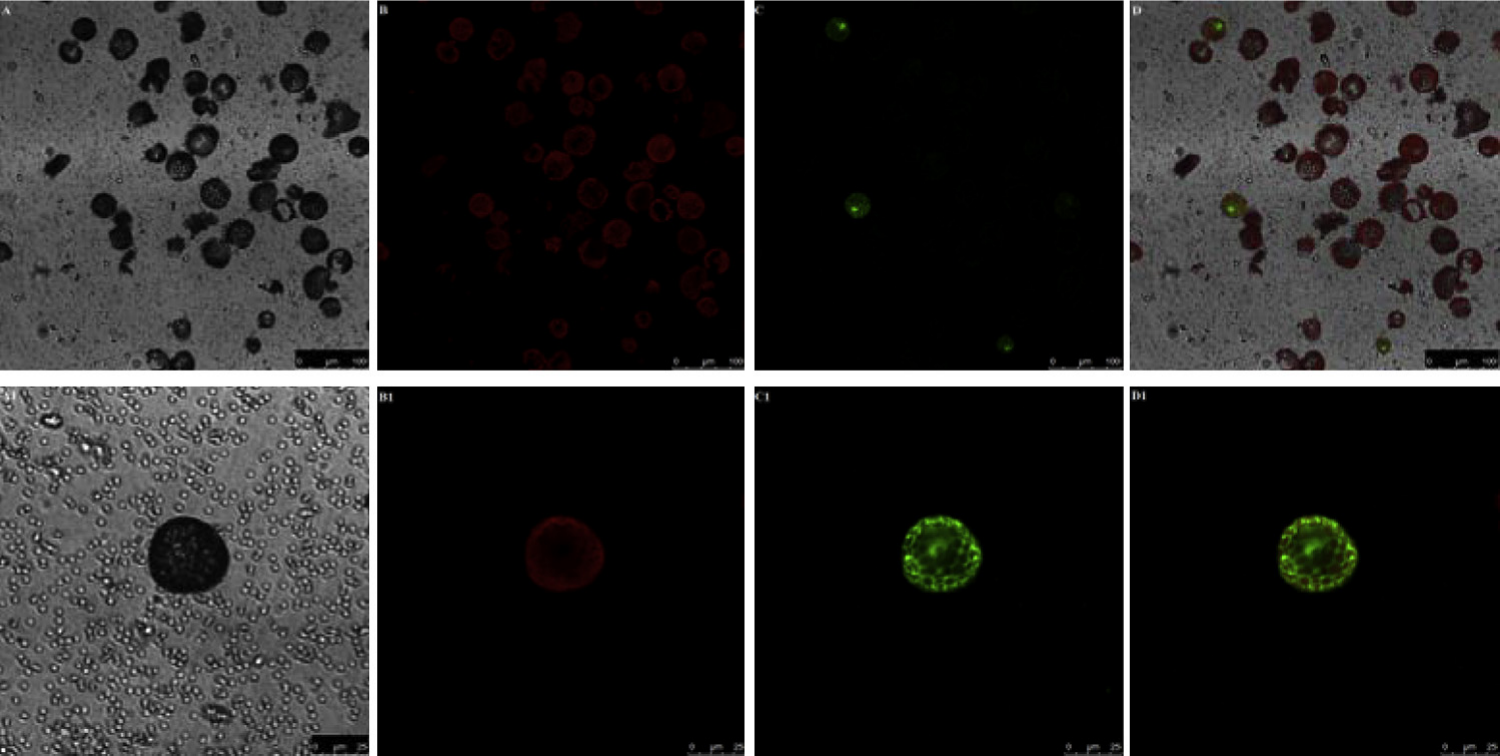
Microscopic picture of immobilized protoplasts for transient gene expression. (A,A1) Bright field images of tobacco protoplasts; (B,B1) autofluorescence of chloroplasts; (C,C1) (D) merged images of A–C.

The confocal microscopic pictures of immobilized protoplasts used for transient gene expression are shown in Fig. 4. The transfection efficiency is low due to many factors, such as the selection of leaves, the quality of protoplasts, the quality of plasmid DNA, the concentration of plasmid DNA, protoplast/DNA ratio, and PEG solution. Therefore, all of these factors should be systemically examined to find the optimal condition.

## Additional information

### Background

Protoplasts are the living material of a plant or bacterial cell, which include the protoplasm and plasma membrane after the cell wall is removed. Due to removal of the cell wall, plant protoplasts have been widely used for genetic transformation, cell fusion [5] and somatic mutation for generating unique and novel plants [3], [6], [7], [8]. Protoplasts obtained through enzymatic cell wall digestion are highly vacuolated and fragile and are easy to rupture during the following experimental steps, such as washing, isolation, and transfection, making it difficult for long-term culture and monitoring of biological processes. Therefore, it is necessary to protect isolated protoplasts from disruption by immobilization.

Polymers prepared from natural or biocompatible gelling agents, such as agarose [9], [10], gellan-gum [11], and alginate [2], [3], [4], [12], are often used as the supporting matrices for cells. Alginate is most popular because the chelation between Na-alginate and calcium chloride does not require high temperature and organic solvents. Meanwhile, Ca-alginate can be conveniently de-polymerized by potassium citrate, releasing encapsulated cells without damage. Although alginate has been widely used for the entrapment of protoplast in the form of beads [13], [14], [15], [16], [17], [18], [19], [20] and film [2], [3], [4], [12], it is not firm enough to provide a good protection for entrapped cells [10], especially when the experimental procedure needs repeated washing and cell collecting. Because silica is chemically inert, optically transparent, biologically resistant to microbial attack, mechanically strong, thermally stable and versatile in shape and size, silica-based matrices have been successfully used to directly encapsulate plant cells [21], [22], [23], [24] or with a multiple step procedure [25], [26], [27] in recent years. For the direct immobilization of plant cell, the colloidal silica interacts strongly with cell walls [27]. For the two-step procedure, cells are first immobilized in Ca-alginate beads that are subsequently trapped in silica matrix, so that harmful contact between cells and the silica precursors is avoided [25].

However, protoplasts without cell walls are more fragile compared with normal plant cell; therefore, the immobilization materials and procedures should be more carefully designed. Inspired by the above studies, we outline a procedure in this work describing the immobilization of tobacco protoplasts by the formation of a uniform film instead of beads. Moreover, we demonstrate for the first time a combined film strategy with the cell-friendly Transwell instead of the glass slide as the substrate, which greatly reduces mechanical damage to the protoplasts, and facilitates easy washing and transfer of cells as well as easy collection of cell secretion or metabolites.

Specifically, in our two-step procedure, the protoplast is first immobilized within Ca-alginate gel in the form of film on Transwell membrane to make protoplasts evenly distributed, then entrapped by silica sol–gel. After the silica sol–gel solidifies, the Ca-alginate is liquefied by potassium citrate, leaving protoplasts entrapped in the cavity of silica network, and the viability of immobilized protoplasts is checked over several days. This immobilization technique can be used on other plant protoplast by using the appropriate enzyme solution, protoplast medium, washing, and incubation solutions. To maintain protoplast viability, the osmolality and pH must be optimized. Keeping an optimized concentration of mannitol and pH in all of the solutions contacting protoplasts is critical to the viability of protoplast. We had initially tried to use immobilized protoplasts for transient gene expression, and despite the low transfection efficiency, this simple approach in immobilization of protoplasts demonstrates the potential to facilitate easy experimental manipulation in the study of transient gene expression, metabolite production and migration assay. Because there are many factors affecting biological processes, all further studies should be systematically examined to determine optimal conditions in order for a good application.

## References

[1] Wu H., Liu W., Tu Q., Song N., Li L., Wang J., Wang J. (2011). Alternate culture and chemical-induced fusion of tobacco mesophyll protoplasts in a microfluidic device. Microfluid Nanofluidics.

[2] Pati P.K., Sharma M., Ahuja P.S. (2005). Alternate extra thin alginate film: an efficient technique for protoplast culture. Protoplasma.

[3] Kielkowska A., Adamus A. (2012). Alternate an alginate-layer technique for culture of *Brassica oleracea* L. protoplasts. In Vitro Cell Dev. Biol.-Plant.

[4] Pati P.K., Sharma M., Ahuja P.S. (2008). Alternate rose protoplast isolation and culture and heterokaryon selection by immobilization in extra thin alginate film. Protoplasma.

[5] Hain R., Stabel P., Czernilofsky A.P., Steinbiss H.H., Herreraestrella L., Schell J. (1985). Alternate uptake integration, expression and genetic transmission of a selectale chimaeric gene by plant-protoplasts. Mol. Gen. Genet..

[6] Karamian R., Ranjbar M. (2011). Alternate Somatic embryogenesis and plantlet regeneration from protoplast culture of Muscari neglectum Guss. Afr. J. Biotechnol..

[7] Sinha A., Caligari P.D.S. (2005). Alternate enhanced protoplast division by encapsulation in droplets: an advance towards somatic hybridisation in recalcitrant white lupin. Ann. Appl. Biol..

[8] Korlach J., Zoglauer K. (1995). Alternate developmental patterns during direct somatic embryogenesis in protoplast cultures of European larch (*Larix decidua* Mill). Plant Cell Reports.

[9] Shillito R., Paszkowski J., Potrykus I. (1983). Alternate agarose plating and a bead type culture technique enable and stimulate development of protoplast-derived colonies in a number of plant species. Plant Cell Reports.

[10] Ripp S., Nivens D.E., Ahn Y., Werner C., Jarrell J., Easter J.P., Cox C.D., Burlage R.S., Sayler G.S. (2000). Alternate controlled field release of a bioluminescent genetically engineered microorganism for bioremediation process monitoring and control. Environ. Sci. Technol..

[11] Nakano M., Hosokawa K., Oomiya T., Yamamura S. (1995). Alternate plant regeneration from protoplasts of *Gentiana* by embedding protoplasts in gellan gum. Plant Cell Tissue Organ Cult..

[12] Dovzhenko A., Bergen U., Koop H.U. (1998). Alternate thin-alginate-layer technique for protoplast culture of tobacco leaf protoplasts: shoot formation in less than two weeks. Protoplasma.

[13] Liu H.B., Kawabe A., Matsunaga S., Murakawa T., Mizukami A., Yanagisawa M., Nagamori E., Harashima S., Kobayashi A., Fukui K. (2004). Alternate Obtaining transgenic plants using the bio-active beads method. J. Plant Res..

[14] Sone T., Nagamori E., Ikeuchi T., Mizukami A., Takakura Y., Kajiyama S.I., Fukusaki E.I., Harashima S., Kobayashi A., Fukui K. (2002). Alternate a novel gene delivery system in plants with calcium alginate micro-beads. J. Biosci. Bioeng..

[15] Ebrahimzadeh H., Noori-Daloii M., Karamian R. (2001). Alternate shoot regeneration from saffron protoplasts immobilized in Ca-alginate beads. J. Sci. Islamic Republic Iran.

[16] Roger D., David A., David H. (1996). Alternate immobilization of flax protoplasts in agarose and alginate beads – correlation between ionically bound cell-wall proteins and morphogenetic response. Plant Physiol..

[17] Kageyama Y., Honda Y., Sugimura Y. (1995). Alternate plant regeneration from patchouli protoplasts encapsulated in alginate beads. Plant Cell Tissue Organ Cult..

[18] Niedz R.P. (1993). Alternate culturing embryogenic protoplasts of ‘Hamlin’ sweet orange in calcium alginate beads. Plant Cell Tissue Organ Cult..

[19] Larkin P.J., Davies P.A., Tanner G.J. (1988). Alternate nurse culture of low numbers of *Medicago* and *Nicotiana* protoplasts using calcium alginate beads. Plant Sci..

[20] Mbanaso E.N.A., Roscoe D.H. (1982). Alternate alginate: an alternative to agar in plant protoplast culture. Plant Sci. Lett..

[21] Meunier C.F., Rooke J.C., Hajdu K., Van Cutsem P., Cambier P., Leonard A., Su B.L. (2010). Alternate insight into cellular response of plant cells confined within silica-based matrices. Langmuir.

[22] Meunier C.F., Dandoy P., Su B.L. (2010). Alternate encapsulation of cells within silica matrixes: towards a new advance in the conception of living hybrid materials. J. Colloid Interface Sci..

[23] Meunier C.F., Rooke J.C., Leonard A., Van Cutsem P., Su B.L. (2010). Alternate design of photochemical materials for carbohydrate production via the immobilisation of whole plant cells into a porous silica matrix. J. Mater. Chem..

[24] Pressi G., Dal Toso R., Dal Monte R., Carturan G. (2003). Alternate production of enzymes by plant cells immobilized by sol–gel silica. J. Sol–Gel Sci. Technol..

[25] Perullini M., Rivero M.M., Jobbagy M., Mentaberry A., Blimes S.A. (2007). Alternate plant cell proliferation inside an inorganic host. J. Biotechnol..

[26] Campostrini R., Carturan G., Caniato R., Piovan A., Filippini R., Innocenti G., Cappelletti E.M. (1996). Alternate immobilization of plant cells in hybrid sol–gel materials. J. Sol–Gel Sci. Technol..

[27] Kuncova G., Podrazky O., Ripp S., Trogl J., Sayler G.S., Demnerova K., Vankova R. (2004). Alternate monitoring of the viability of cells immobilized by sol–gel process. J. Sol–Gel Sci. Technol..

